# Quantitative Model of Cell Cycle Arrest and Cellular Senescence in Primary Human Fibroblasts

**DOI:** 10.1371/journal.pone.0042150

**Published:** 2012-08-07

**Authors:** Sascha Schäuble, Karolin Klement, Shiva Marthandan, Sandra Münch, Ines Heiland, Stefan Schuster, Peter Hemmerich, Stephan Diekmann

**Affiliations:** 1 Research Group Theoretical Systems Biology, Friedrich-Schiller-University, Jena, Germany; 2 Department of Bioinformatics, Friedrich-Schiller-University, Jena, Germany; 3 FLI, Beutenbergstr. 11, Jena, Germany; University of Edinburgh, United Kingdom

## Abstract

Primary human fibroblasts in tissue culture undergo a limited number of cell divisions before entering a non-replicative “senescent” state. At early population doublings (PD), fibroblasts are proliferation-competent displaying exponential growth. During further cell passaging, an increasing number of cells become cell cycle arrested and finally senescent. This transition from proliferating to senescent cells is driven by a number of endogenous and exogenous stress factors. Here, we have developed a new quantitative model for the stepwise transition from proliferating human fibroblasts (P) *via* reversibly cell cycle arrested (C) to irreversibly arrested senescent cells (S). In this model, the transition from P to C and to S is driven by a stress function γ and a cellular stress response function F which describes the time-delayed cellular response to experimentally induced irradiation stress. The application of this model based on senescence marker quantification at the single-cell level allowed to discriminate between the cellular states P, C, and S and delivers the transition rates between the P, C and S states for different human fibroblast cell types. Model-derived quantification unexpectedly revealed significant differences in the stress response of different fibroblast cell lines. Evaluating marker specificity, we found that SA-β-Gal is a good quantitative marker for cellular senescence in WI-38 and BJ cells, however much less so in MRC-5 cells. Furthermore we found that WI-38 cells are more sensitive to stress than BJ and MRC-5 cells. Thus, the explicit separation of stress induction from the cellular stress response, and the differentiation between three cellular states P, C and S allows for the first time to quantitatively assess the response of primary human fibroblasts towards endogenous and exogenous stress during cellular ageing.

## Introduction

Ageing is an omnipresent process observed throughout all organisms, yet its fundamental driving forces remain unclear. Some aspects of ageing are believed to be recapitulated during cellular senescence of some types of primary mammalian cells in cell culture systems [1]. Notably, experimental clearance of cellular senescent cells in mice delays ageing-related pathologies in at least some tissues [2]. *In vitro*, cellular senescence manifests as a permanent cell cycle arrest resulting from the replicative exhaustion of cultured normal diploid cells [3]. Senescence also acts as an efficient tumor suppressor mechanism [4].

Although senescent cells are unable to proliferate, they are still viable and metabolically active, but resistant to mitogenic or apoptotic stimuli [5], [6]. To identify senescent cells *in vitro* and *in vivo*, specific biomarkers have been established [7], [8] reviewed in [9], [10]. Senescent cells are characterized by an increased cell size associated with higher numbers of lysosomes, vacuoles and mitochondria, and major changes in the cytoskeleton (reviewed in [11]). Diagnostically important senescence markers include a high activity of senescence-associated β-galactosidase (SA-β-Gal) [8], telomere dysfunction-induced foci (TIF) [12], [13] as well as up-regulation of the cell cycle regulators p16, p21 and p53 [14], [15]. Many, but not all primary human cell types develop senescence associated heterochromatin foci (SAHF) [7], [16]. Furthermore, senescent cells express matrix-degrading proteases and inflammatory chemokines and cytokines, known as the senescence-messaging secretome (SMS) or senescence-associated secretory phenotype (SASP) [17]–[21].

Several mechanisms are essential for the induction of senescence including an intact DNA damage response and functional p53-p21, p16-pRb, and p38-MAPK pathways [9]. Telomere-dependent cellular senescence is induced by an increased DNA damage response (DDR)-activity at dysfunctional telomeres [12]. Uncapped telomeres focally accumulate high levels of phosphorylated H2AX and repair factors such as p-ATM, 53BP1, MDC1, and pNBS1 [12], [13], [22]–[24]. Activated ATM at dysfunctional telomeres induces the downstream effectors Chk1 and Chk2. These kinases activate p53, which induces transcription of p21 to promote cell cycle arrest [12], [25], [26], reviewed in [27]–[30]. While cells with damaged telomeres always activate the p53-p21-pathway, the p16-pRb-pathway alone is not sufficient to trigger DNA damage-induced senescence [12]. DNA damaging interventions such as irradiation or drug treatment induce the same cascade of responses if DNA damage exceeds a certain threshold [15], [31]–[39]. Overexpression of activated oncogenes, e.g. RAS, induces oncogene-induced senescence (OIS) [35], [40], [41], probably also through signaling from hyperproliferation-induced DNA damage sites, in this case at collapsed replication forks [35], [42]–[44]. OIS appears to be mainly dependent on the p16-pRb-pathway [45]. Consistent with these observations, freshly isolated normal human epidermal fibroblasts are resistant to RAS-induced senescence because of their low or absent levels of p16 [46]. In general, cellular senescence is considered to be a stress-response program which can be activated by various stressors most prominently by excessive DNA damage and telomere shortening [47].

Cultured senescent cells can be easily identified by the absence of proliferation (Ki67-negativity) combined with the identification of DNA damage foci. Additionally, in particular when being combined with the two markers, SA-β-Gal activity is a good quantitative indicator of senescence [48]. SA-β-Gal activity is detectable at high levels in senescent, but not in pre-senescent or quiescent mammalian cells in culture [8]. Furthermore, the marker is undetectable in immortal cell lines, yet is induced when cells are growth-arrested by genetic manipulations [8]. Recently, we have identified accumulation of annexin A5 at the nuclear envelope as an additional robust and quantitative marker for cellular senescence [49].

With increased age, cell populations, either in tissues or in culture, will be a mixture of proliferating, cell cycle arrested and senescent cells [50], [51]. Cell cycle inhibitors, in particular p21 and p16, diagnostically indicate the presence of arrested human fibroblast cells. On the other hand, robust senescence markers such as SA-β-Gal identify senescent cells. In parallel, growth curves of cell populations yield the information on total cell number so that at any time point, three cellular states can be estimated: proliferating, cell cycle arrested and senescent cells. This enables the establishment of quantitative computer models describing cellular growth and the transition to senescence. Here we present such a model, depict its properties and apply the new model to the analysis of growth rates. The cellular response to low dose irradiation (low stress) driving primary cells into cell cycle arrest without senescence, can be quantitatively described by the model. At high dose irradiation, higher population doublings and thus higher stress levels, cells become senescent, a transition also well described by the model. From the quantitative fit of the growth curves by our model, the population of the arrested C and senescent S states can be estimated and compared to experimental values. We also employed the model to quantitatively describe subtle differences in the cellular ageing process of different primary human fibroblasts.

## Materials and Methods

### Cell Lines

Primary human fibroblasts MRC-5 (primary cells, *Homo sapiens*, 14 weeks gestation male, fibroblasts from normal lung, normal diploid karyotype), WI-38 (primary cells, *Homo sapiens*, 3 months gestation female, fibroblasts from normal lung, normal diploid karyotype) and BJ (primary cells, *Homo sapiens*, newborn male, fibroblasts from normal foreskin, normal diploid karyotype), IMR-90 (primary cells, *Homo sapiens*, fibroblasts from normal lung, normal diploid karyotype) were obtained from ATCC (LGC Standards GmbH, Wesel, Germany). HFF (primary cells, *Homo sapiens*, fibroblasts from foreskin, normal diploid karyotype) cells were kind gifts of T. Stamminger (University of Erlangen, Kronschnabl and Stamminger [52]).

### Cell Culture

Cells were cultured as recommended by ATCC in Dulbeccos modified Eagles low glucose medium (DMEM) with L-glutamine (PAA Laboratories, Pasching, Austria), supplemented with 10% fetal bovine serum (FBS) (PAA Laboratories). Cells were grown under normal air conditions in a 9.5% CO_2_ atmosphere at 37°C. For sub-culturing, the remaining medium was discarded and cells were washed in 1xPBS (pH 7.4) (PAA Laboratories) and detached using trypsine/EDTA (PAA Laboratories). Primary fibroblasts were sub-cultured in a 1∶4 ( = 2 PDs) or 1∶2 ( = 1 PD) ratio.

### Immuno-fluorescence

Analysis of senescence (p21, p16) and heterochromatin markers (recognizing SAHFs) as well as the DNA damage indicator γH2AX were determined by immuno-fluorescence at the single cell level. For this analysis in young and senescent cells, cells were fixed in 4% paraformaldehyde (in 1xPBS, pH 7.4) for 10 min. Cells were permeabilisized using 0.25% Triton-X 100 (in 1xPBS, pH 7.4) for 3 min. Primary antibodies (anti-p21 (H-164, Santa Cruz), anti-p16 (BD Pharmingen), anti-γH2AX (16–193, Upstate, USA)) were diluted in 1xPBS (pH 7.4) and incubated on the cells for 1 hr at RT. SAHFs (“dense DAPI regions”) were visualized either by DAPI staining or by immune-fluorescence using anti-bodies against H3K9me3 (ab8898, Abcam, Cambridge, UK) or H4K20me3 (ab9053, Abcam). The secondary fluorescently labeled antibodies were incubated for 1 hr at RT. The DNA was stained with 4′-6-diamidine-2-phenyl indole (DAPI, Invitrogen, Carlsbad, USA) by mounting the slides in Prolong gold antifade reagent (Invitrogen). Cells were imaged using a Zeiss LSM 510 or LSM 710 microscope with a 63x/1.40 oil immersion objective. Immuno-fluorescence results were obtained by determining the number of cells positive for the respective marker in relation to the total cell number. 100 cells, identified by DAPI-stained nuclei, were visually investigated by fluorescence microscopy in each experiment. Marker-positive cells were identified by detecting the specific fluorescence-labeled antibodies, cells without the respective marker did not show fluorescence. Mean values and standard deviations represent a minimum of three independent experiments.

### Induction of Cellular Senescence

Cellular senescence was induced by γ-irradiation: human fibroblast cells were irradiated by ionizing radiation in a Gammacell GC40 (MDS Nordion, Ottawa, Canada) by ^137^Cs as radioactive isotope. Exposure time was determined by correcting the irradiation dose of 1.23 Gy/min with decay time. Cells were seeded 1 day before exposure, irradiated at RT, and subsequently cultured at 37°C.

### Detection of SA-β-galactosidase

The SA-β-galactosidase (SA-β-Gal) assay was performed as described by Dimri et al. [8]. Cells were washed in 1xPBS (pH 7.4) and fixed in 4% paraformaldehyde (pH 7.4), 10 min at RT. After washing the cells in 1xPBS (pH 7.4), staining solution consistent of 1 mg/ml X-Gal, 8 mM citric acid/sodium phosphate pH 6,0, 5 mM K_3_Fe(CN)_6_, 5 mM K_4_Fe(CN)_6_, 150 mM NaCl, 2 mM MgCl_2_, was added. The enzymatic reaction occurred without CO_2_ for 4–16 hours at 37°C. After incubation the cells were washed in 1xPBS (pH 7.4) and, in order to visualize cell nuclei, DNA and SAHFs, mounted with DAPI-containing Prolong Gold antifade reagent (Invitrogen).

### Model Simulation

We simulated our model of cell cycle arrest and cellular senescence as a system of ordinary differential equations using standard ODE solvers from the MATLAB environment (http://www.mathworks.com; Natick, USA). For simplicity, linear kinetics were chosen for the transition between different cell states. Experimental data were fitted by the model using MATLABs lsqnonlin function. The quality of the fit was evaluated by solving the least squares optimization problem.

### Model Adjustments for γ-irradiation Induced DNA Damage

After a stress situation, cells will always continue to proliferate if a single functional cell survived. We thus considered a cell concentration below one as growth termination criteria in our simulation. For high irradiation doses, cells are critically damaged and transformed into a senescent cell state, with the number of proliferating cells reduced below one. However, if cells are only slightly damaged by a low irradiation dose, proliferating cells survived and the cell population recovered.

**Figure 1:**
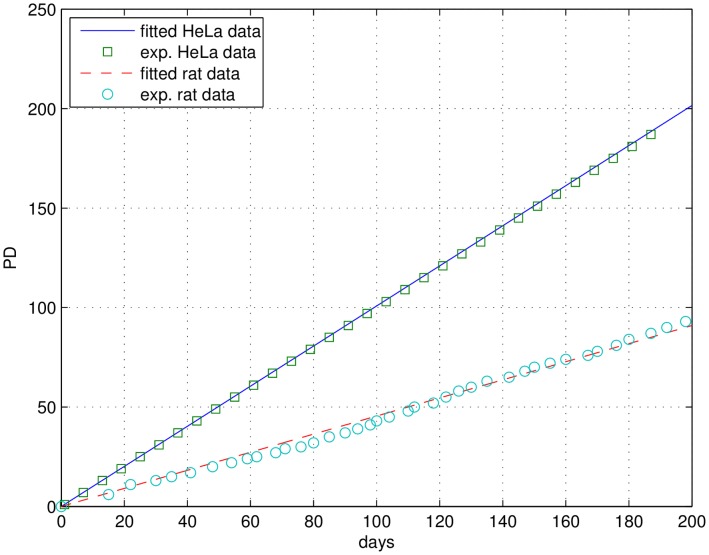
Fit of Eq. 1 to constant growth for HeLa (own data: green squares, fit: blue line) and rat fibroblast cells (data: blue circles [**53**], fit: red dashed line). See also Table 1.

**Figure 2:**
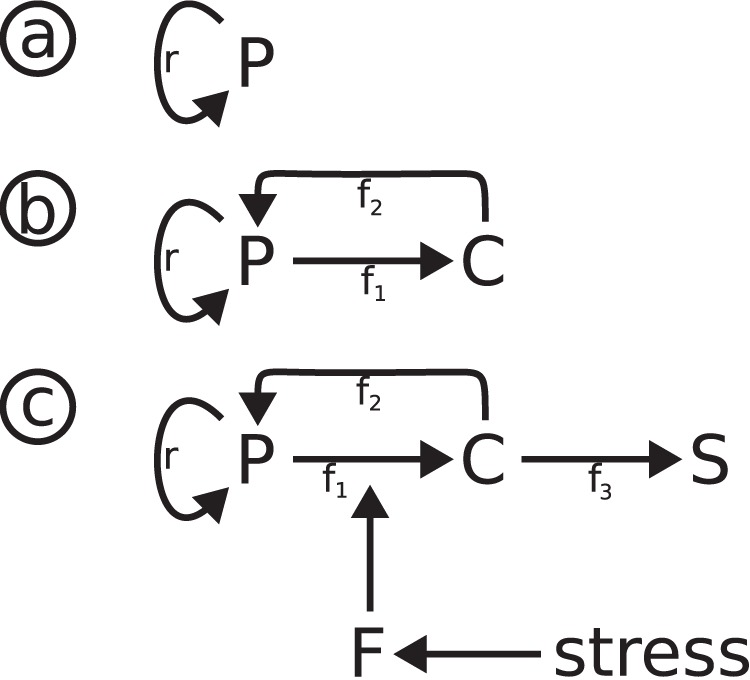
Model scheme. a, proliferation; b, extension with second cell state species; c, complete final model (upper part: P-C-S transitions; lower part: stress induction and cell response function F).

## Results

### Model Deduction

#### Constant growth

A number of immortal cell lines show constant and stable growth (Figure 1). Freshly isolated primary fibroblasts share this feature during the early periods of their lifespan, although their growth rate can be lower. In a most simple approach, this situation can be described by a cell population solely consisting of proliferating cells (Figure 2A). With a population of healthy, proliferating cells, P(t), constantly growing with time t, the time-dependent change of P(t) depends on the constant growth factor *r* (Eq. 1):


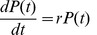
(1)

Eq. 1 results in exponential growth and yields in a linear graph when plotting population doublings (PD) versus time (Figure 1). Fitting this minimal model to experimental data on various rodent species from the literature [53] and our own data (HeLa cells), starting with a single cell, we derive unique division rates r for different cell lines which vary up to a factor of 7.8 between minimal and maximal growth rates (Table 1). Cells not only from different species but also cells from the same species but at different age (e.g. growth rates of adult versus embryonic squirrel fibroblasts, see Table 1) show a significant difference in their unhampered constant growth speed. The fastest growth rate was measured for the cancer cell line HeLa which is supposed to be made possible by neglecting cellular maintenance [54]–[56].

**Table 1:** Constant growth, model parameter r fitted to experimental data.

Species	Parameter *r*
House mouse	0.28
Embryonic squirrel	0.20
Squirrel	0.09
Naked mole rat	0.16
Gerbil	0.25
Chinchilla	0.13
Rat	0.32
Chipmunk	0.13
Guinea pig	0.26
Muskrat	0.15
Hamster	0.28
HeLa	0.70

Experimental data from the literature (rodent species [53]) and our own data (HeLa cells).

#### Complete model

**Figure 3:**
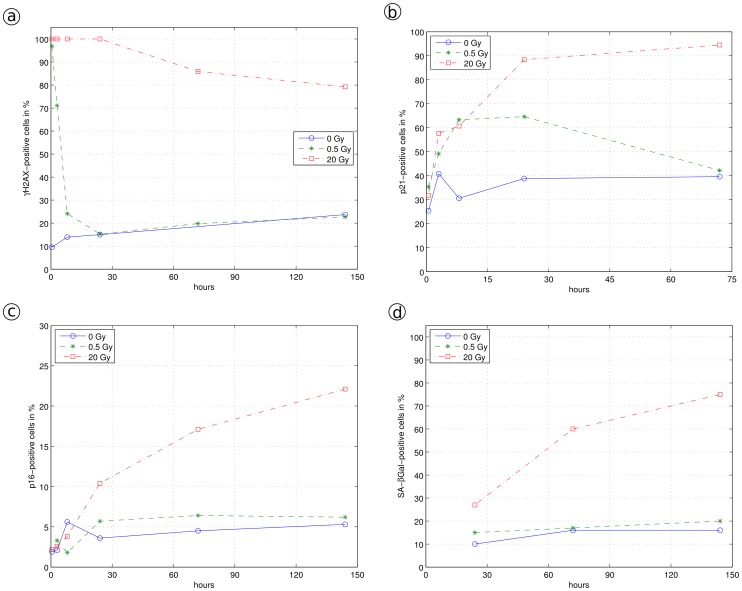
Relative number MRC-5 fibroblast cells positive for cellular markers after irradiation by the doses 0, 0.5 and 20 Gy. MRC-5. a, DNA damage marker γH2AX; b, cell cycle arrest marker p21; c, cycle arrest marker p16; d, senescence marker SA-β-Gal. The experimental error in such experiments is less than ±5%.

**Figure 4:**
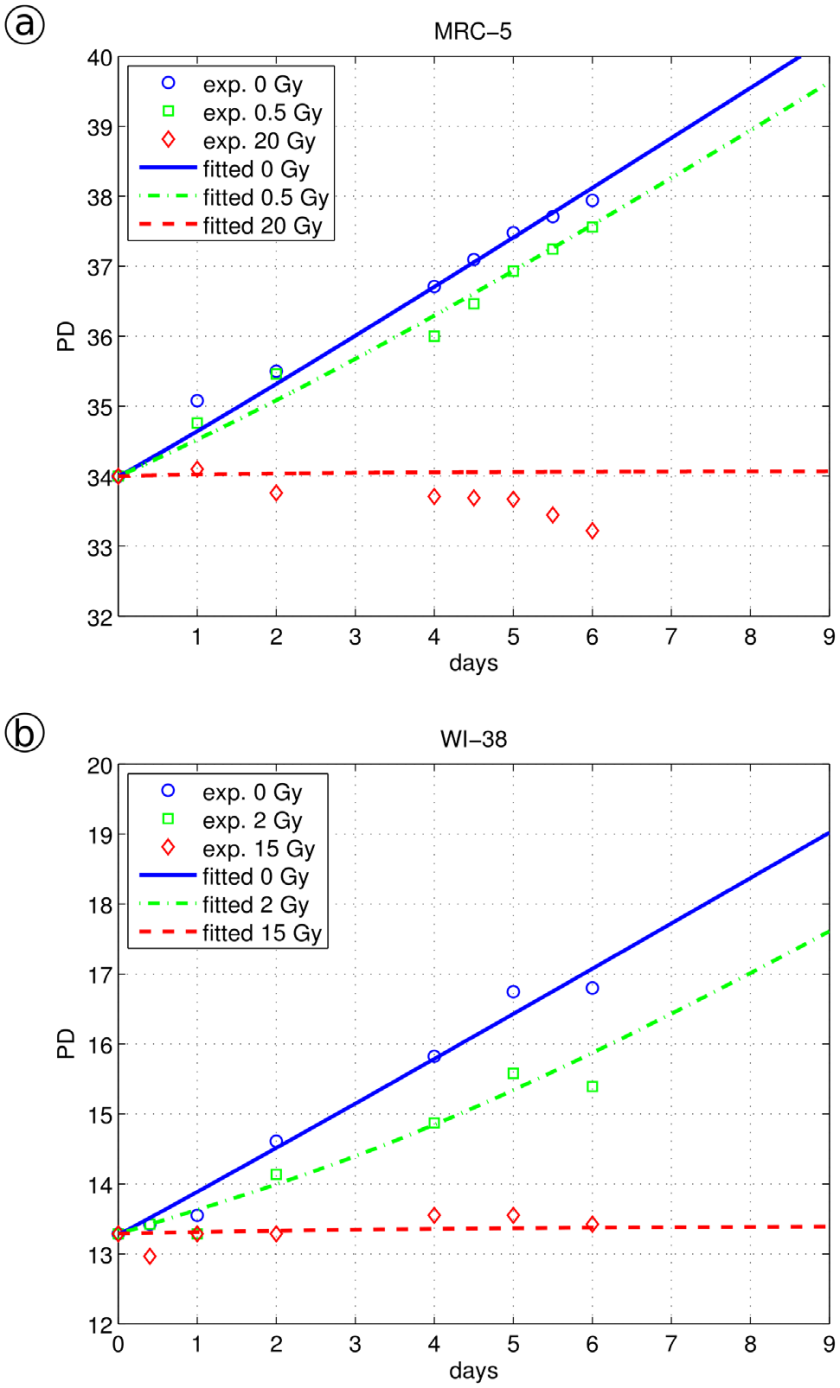
PD curves of human fibroblasts. Model fitting of different radiation doses and experimentally derived PDs. The data were fitted using the same parameter set for all radiation doses (differing only in the applied amount of irradiation time) applying the model described by Eq. 3a–d: a, MRC-5 fibroblast, different radiation doses 0, 0.5 and 20 Gy; b, WI-38 fibroblast, radiation doses 0, 2 and 15 Gy.

Mild stress can lead to short term reversible cell cycle arrest [57], [58]. We quantitatively analyzed this effect by irradiating MRC-5 fibroblasts with 0.5 Gy, inducing low levels of DNA damage as indicated by increased numbers of γH2AX DNA repair foci determined using immuno-fluorescence (Figure 3A). Within 16 hrs after irradiation, the number of p21-positive cells (determined by immuno-fluorescence) increased indicating short term cell cycle arrest (Figure 3B); within the following hours, the number of p21-positive cells decayed to background levels (73 hrs) indicating successful DNA repair and return into the cell cycle. This low dose irradiation did neither result in an increase of the number of p16-positive cells (investigated using immuno-fluorescence, Figure 3C) nor in the up-regulation of the cellular senescence marker SA-β-Gal (percentage of SA-β-Gal positive cells, Figure 3D). The cell population continued to grow with a slight time lag, consistent with cell cycle re-entry after a transient cell cycle arrest (Figure 4A). In order to quantitatively describe this reversible cell cycle arrest, we introduced the additional cell cycle state “C” (Figure 2B) with the rate f_1_ for the transition from proliferating cells P to cell cycle arrested cells C and the rate f_2_ for the transition back into the cell cycle. This model can be described by the following equations:


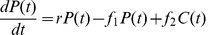
(2a)


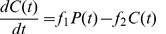
(2b)

After high dose irradiation, MRC-5 (20 Gy) (or WI-38 (15 Gy)) fibroblasts showed a very different response (Figure 3A–D). Immediately after irradiation, all cells had a highly elevated number of γH2AX repair foci which decreased only slightly within the following 6 days (Figure 3A). The number of p21, and now also the number of p16-positive cells increased after irradiation, associated with a complete stop of cell proliferation and a constant increase of the number of SA-β-Gal-positive cells (Figure 3B, C, D). SA-β-Gal, a quantitative marker of cellular senescence [8], [48], indicates the presence of an increased number of cells irreversibly arrested in the cell cycle. This senescent phenotype extends our model by an additional state “S” which is populated by rate f_3_ from the C state (Eq. 3c); S cannot be derived directly from the P state, nor can it return into the C or P state (no back reaction, Figure 2C, upper part).

The simple model (Eq. 2a, b) with time-independent parameters f_1_ and f_2_ describes an immediate response to mild stress. However, we only observed an immediate response for the appearance of γH2AX repair foci (Figure 3A). For other markers, we observed a slightly delayed response, with p21 levels reaching maximal levels about 16 hrs after irradiation when γH2AX DNA damage foci already had declined close to background levels (Figure 3A, B). We thus have to consider a delayed cellular response to stress. Furthermore, the transition from reversibly (C) to irreversibly (S) arrested cells depends on the level of irradiation, or, in general, on the stress level applied to the cells. Thus, the P-C-S transition model presented in Figure 2C (upper part) must be extended to include a stress factor and a cellular stress response function F, sensing this difference: F allows for continued population growth under mild stress, but emphasizes the transition to the senescent phenotype when faced with harsh stress, resulting ultimately in replicative senescence (Figure 2C). This model can be described by the equations:


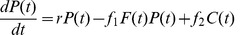
(3a)


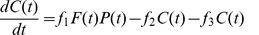
(3b)


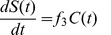
(3c)


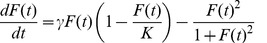
(3d)

The stress response function F(t) (Eq. 3a, b, d), multiplied by the stress expression γ, describes the influence of cellular stress phenomena, such as exposure to irradiation, oxidative stress, telomere shortening, etc. While the external stress itself can be short, function F describes the cellular response, which can be delayed relative to the various stresses: function F can “remember” that a stress event has occurred, as its value might not decrease rapidly, once the stress itself has disappeared.

Function F, adapted from Ludwig et al. [59] was chosen to have a bi-stable behavior (Eq. 3d): below a certain stress threshold, the F value is low (resembling a healthy cell), but F rapidly turns to high values once this threshold is exceeded (resembling an endangered cell). Once the stress function γ dropped below threshold values, F decreases slowly, describing a slow return to the normal cell state. Indeed, a bifurcation analysis confirmed that function F, chosen in this form, is bi-stable and, depending on parameter values, switches between two states (Supplement S1). F is defined by two parameters, K and γ. Whereas K is the maximal value F can reach, γ denotes various forms of stress, ranging from sudden stress such as irradiation pulses, to accumulating stress resulting e.g. from telomere shortening or non-physiological oxygen concentrations which represent long term effects. When irradiation is applied, γ>0 for the time cells are exposed to irradiation, and γ = 0 otherwise. In contrast, when simulating long-term stress, we substituted a slowly increasing function for γ in order to resemble accumulating effects (see below), according to Equation 4:


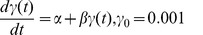
(4)

Introducing a stress (γ) and a response (F) function separately, offers the advantage that the effect of any kind of stress and the cell-specific response to this stress can be modeled individually or in selected combinations, as large variations had been observed [60], and cells from different tissue origin might respond differently [46]. Here we analyzed cells from the same tissue source (lung; MRC-5, WI-38), the same donor age (MRC-5, WI-38, BJ) and the same/different gender (male: MRC-5, BJ; female: WI-38).

#### Sensitivity analysis

We investigated the sensitivity of the model parameter values regarding the resulting PD curves and cell state distributions in the culture (Supplement S2). The analysis clearly showed that our model is most sensitive to the variation of the growth rate r (resembling its particular importance for the maximal replicative capacity of the fibroblast cells) and much less sensitive to the other parameter f_1–3_, K, α and β. Interestingly, WI-38 cells are notably less sensitive to f_2_ and f_3_ variation than BJ cells.

### Model Application to Differently Stressed Cells

#### Irradiation induced DNA damage in human fibroblast WI-38 cells with 0, 2, or 15 Gy

Primary human WI-38 fibroblasts were γ-irradiated with 2 Gy or 15 Gy inducing low or high levels of DNA damage, respectively. The cellular stress response was analyzed by immonu-fluorescence before and 0.5 hours, 1 day and 6 days after irradiation [61]. Immediately after irradiation, we observed increased γH2AX levels, which, after low irradiation, decayed to background levels within one day, whereas they decayed to background levels only after three days when irradiated with a high dose. One and 6 days after irradiation we noticed an increase in p21 levels which was only slight after low, but strong after high levels of irradiation, while p16 levels increased only after 6 days and high dose irradiation. The number of SA-β-Gal positive cells within the population increased from low levels (13±3%) for untreated cells to 30±1% and 67±6% after 6 days for 2 Gy and 15 Gy irradiated cells, respectively, as revealed by the SA-β-Gal staining. Compared to untreated cells, low level irradiation delayed growth but cells continued to proliferate after about one day (Figure 4B). In contrast, high irradiation levels resulted in severe DNA damage so that cells stopped proliferation irreversibly (Figure 4B).

Applying our model for stress-induced cell cycle arrest and senescence (Figure 2C), we simulated the irradiation experiment by selecting a value for γ in the range of values 700<γ<6,000. The value of γ is crucial for the quantitative description of the low irradiation growth curve. Without irradiation, we apply here γ = 0, and for strong irradiation, γ exceeds a threshold value and is not well-defined above. For γ<700, we observe that cells are able to recover even from strong irradiation, and cells under mild irradiation are only insignificantly hampered in growth, indicating that its value is set too low. In contrast, for γ>6,000, our simulation does not allow cells suffering from mild irradiation to recover, showing that such high γ values cannot resemble sensitivity of WI-38 cells to irradiation. Consequently, setting γ = 2,800 arbitrary units for the irradiation time (zero for no irradiation, 108 sec for 2 Gy, 13.5 min for 15 Gy), we were able to fit one set of model parameters (r, f_1_, f_2_ and f_3_) to the three different measured growth curves. For the values r = 0.46, f_1_ = 3.8, f_2_ = 16 and f_3_ = 0.26 and three different irradiation times for γ, we obtained a convincing description of the experimental behavior (see Figure 4B).

#### Irradiation induced DNA damage in human fibroblast MRC-5 cells with 0, 0.5, or 20 Gy

A similar irradiation experiment was conducted using MRC-5 cells, γ-irradiated with 0.5 Gy or 20 Gy, as described above (see Figure 3A–D). Applying our model, we successfully fitted the parameter set r = 0.51, f_1_ = 4.0, f_2_ = 16 and f_3_ = 0.42 to the measured growth curves (Figure 4A). In order to resemble irradiation-induced damage, γ was selected within the range of values 800<γ<29,000. For γ<800, we again observed that mildly stressed cells grew almost without delay and that cells suffering from strong irradiation were still able to recover. For γ>29,000, we observed that even low dose irradiated cells were unable to recover, indicating again that such high γ values are outside of the relevant range. We set γ = 0 for no irradiation, γ = 8,000 over 27 sec for 0.5 Gy, and over 18 min for 20 Gy. Choosing the same γ value for the time of irradiation as for WI-38 fibroblasts (γ = 2,800), we were unable to explain the MRC-5 data. Although we could fit well the 20 Gy irradiation data with γ = 2,800, the mildly irradiated MRC-5 cells appeared to be not sufficiently stressed, resulting in a growth rate almost as high as cells that were not irradiated. Nevertheless, both values of γ indicate a rapid change of F to the endangered state, triggering subsequent cell cycle arrest. The different γ values suggest that WI-38 fibroblasts are considerably more sensitive to (this) stress compared to MRC-5 cells (see also [60]), consistent with a lower γ value for the exposure time. Choosing a similar sensitivity for MRC-5 cells (γ values similar to WI-38 levels), would reduce the quality of the fitted curves considerably.

#### “S” feedback on proliferation

Another hallmark of cellular senescence is the formation of a senescence associated secretory phenotype (SASP) which is marked by the secretion of inflammatory cytokines [20], [21], [62], [63]. The secretion of these factors is mainly dependent on an increased and persistent DNA damage response and is therefore specific for senescent cells [64]. These secreted factors might have an effect on the growth behavior of the cell population. To investigate the effect of SASP secreted from “S” cells on the growth behavior (rate r) of proliferating “P” cells and their transition to “C” cells (f_1_, f_2_), early passage MRC-5 cells were cultured in medium harvested from cells which were senescent for at least one week. This senescence-conditioned medium was then constantly kept on the young cells and replaced with fresh senescence-conditioned medium every week. In this preliminary experiment, young cells did not show impaired proliferation compared to control cells. Thus, senescence-conditioned medium did not induce senescence in young passage fibroblasts. Only at very late passages, cells cultured with the senescence-conditioned medium tend to senesce approximately two population doublings earlier than control cells. In a more extensive study, influences of short cytokine half-lives or strong dilution effects should be excluded. Nevertheless, our preliminary data indicate that the presence of “S” cells seem to influence the growth of the whole cell population in its entirety only slightly (“S” seems to have little influence on r, f_1_ and f_2_).

#### Replicative senescence induced by 20% oxygen and telomere shortening

**Figure 5:**
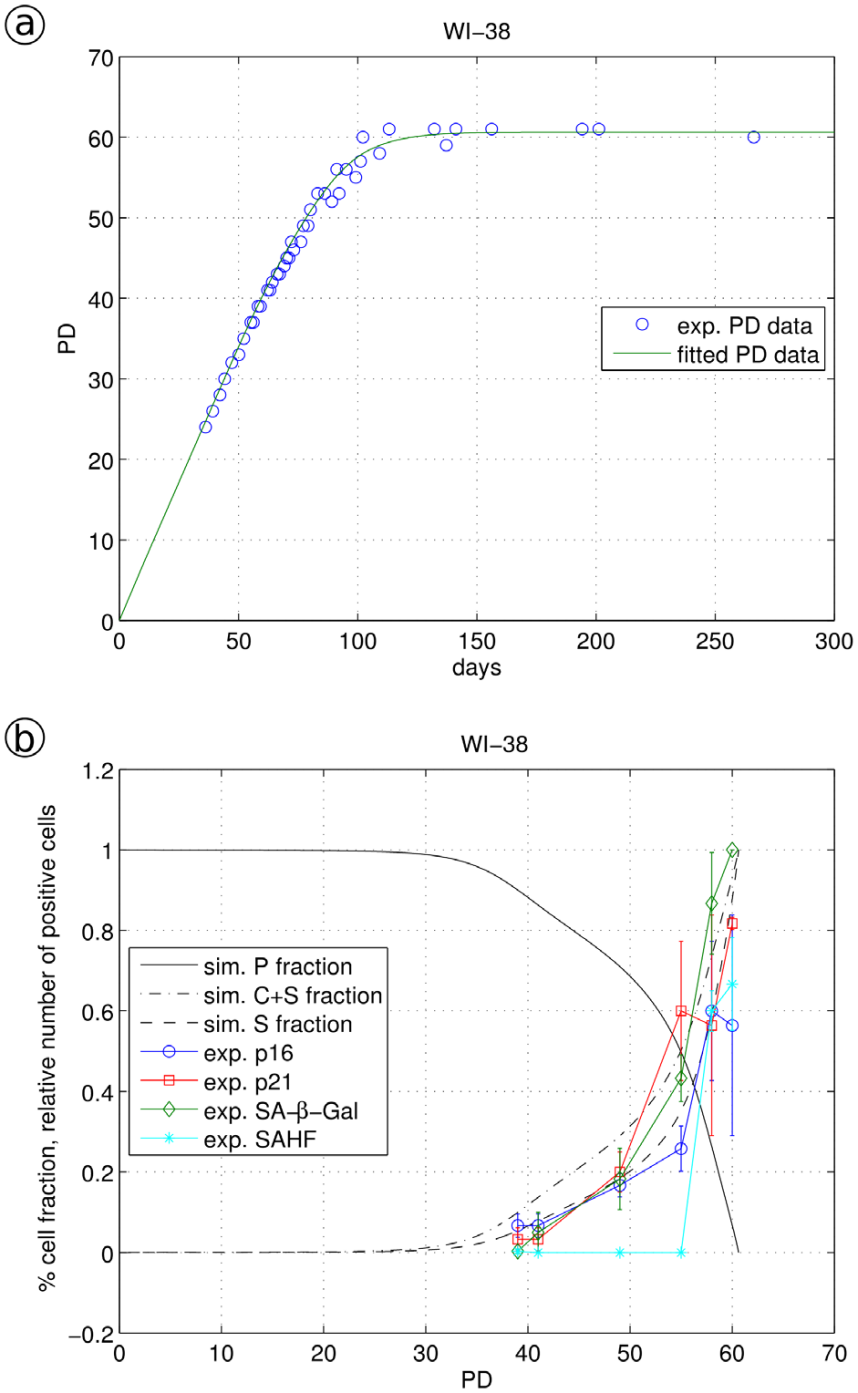
Simulation of WI-38 fibroblast data. a, Experimental growth data (circled) were fitted by model Eq. 3a–d using Eq. 4 as an expression for monotonically increasing stress γ; b, the fraction of proliferating cells P, cells showing a cell cycle arrest or a senescent phenotype (C+S) and solely the fraction of senescent cells S are shown together with the appearance of biomarkers. Biomarker values (p16, p21, SA-β-Gal and SAHF) were measured by immune-fluorescence as number of positive cells [49].

When human fibroblast WI-38, BJ and MRC-5 cells continue to grow, they stop proliferation when reaching their Hayflick limit [3]. This state of replicative senescence may be determined by both, cell-specific stress responses (due to for example constant oxidative stress) and telomere shortening [65]. The growth behavior and the number of cells with up-regulated markers p21, p16, SA-β-Gal and SAHF were determined for MRC-5, WI-38 and BJ cell lines [49]. Immuno-fluorescence analyses of all three cell lines showed, after a certain time in culture, an elevated number of cells with higher levels of p16, p21 and SA-β-Gal. Our studies additionally revealed that senescent cells accumulated persistent DNA damage, as shown by increased γH2AX levels (data not shown) and loss of the proliferation marker Ki67 [49]. WI-38 cells showed a low number of cells with up-regulated markers for p16, p21, SA-β-Gal and SAHFs at PD 40 but high numbers at PD 60 (here, all cells were SA-β-Gal positive), with very little delay between the various markers indicating an immediate transition from proliferation to senescence (see Figure 5B). To quantitatively describe these data, we substituted γ according to Equation 4 using α = 0.002 and β = 0.028 for the description of the long term accumulating stress. With the fitted values r = 0.47, f_1_ = 0.40, f_2_ = 0.43 and f_3_ = 0.66, we obtained a quantitative fit of the WI-38 growth curve (Figure 5A) and the marker increase (Figure 5B).

**Figure 6:**
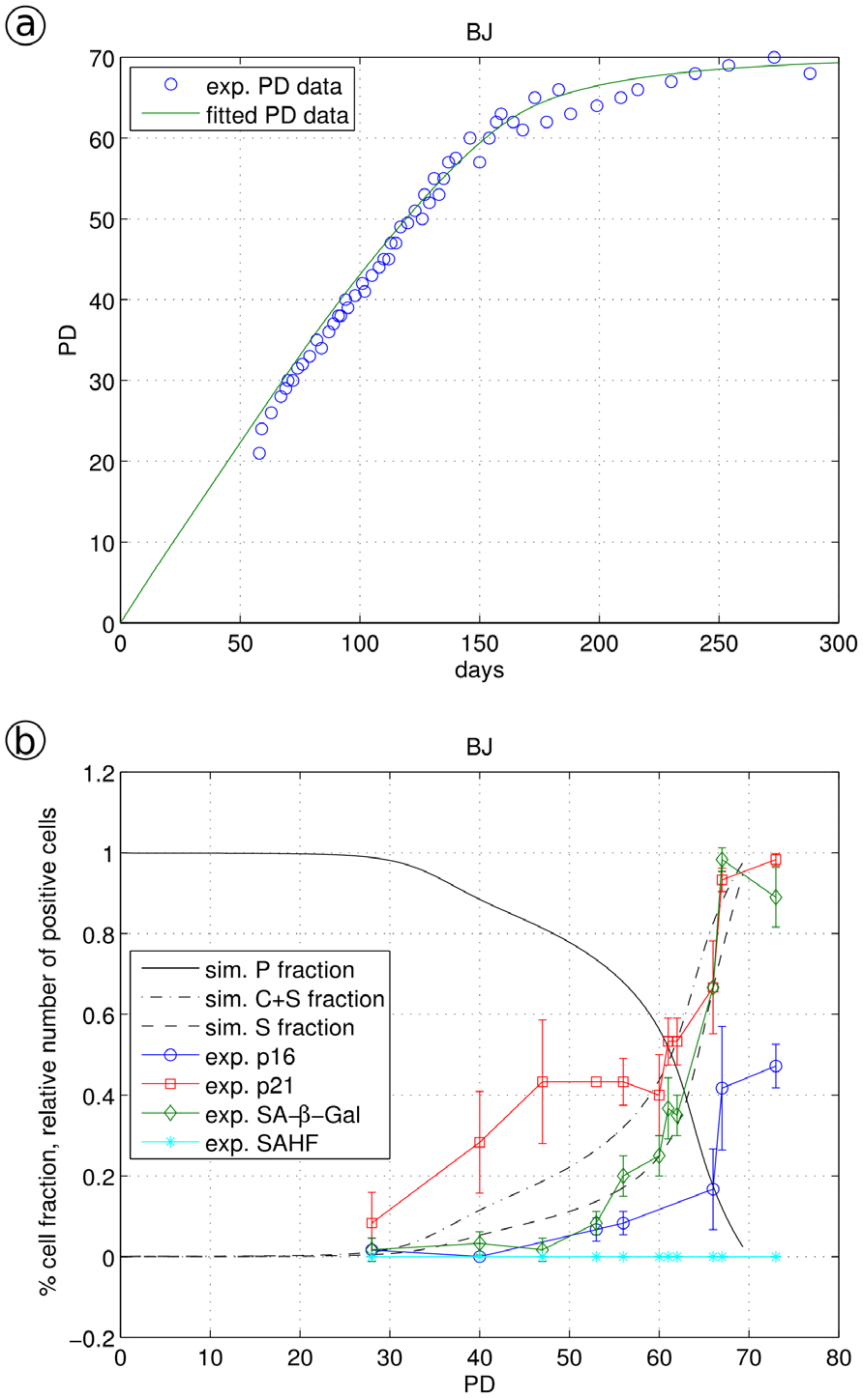
Simulation of BJ fibroblast data. a, experimental data (circled) were fitted by model Eq. 3a–d using Eq. 4 as an expression for monotonically increasing stress γ; b, the fraction of proliferating cells P, cells showing a cell cycle arrest or a senescent phenotype (C+S) and solely the fraction of senescent cells S are shown together with the appearance of biomarkers. Biomarker values (p16, p21, SA-β-Gal and SAHF) were measured by immune-fluorescence as number of positive cells [49].

For BJ fibroblast cells at higher PDs, the timing of the up-regulation of senescence markers varied strongly: p21 levels started to increase already at PD 30 while the number of SA-β-Gal positive cells started to increase at PD 50 (Figure 6B). Even at high PD values, BJ cells did not show SAHF formation. Highly compacted heterochromatin foci, termed senescence associated heterochromatin foci (SAHF), develop during senescence in many, but, as also shown here, not all primary human cell types [7], [16]. Applying γ (Eq. 4) with α = 0.001 and β = 0.016 for the description of long-term accumulating stress and the fitted values r = 0.30, f_1_ = 1.1, f_2_ = 2.45 and f_3_ = 0.27 we obtained a quantitative fit of the BJ growth curve (Figure 6A) with a good fit for p21 and SA-β-Gal increase (Figure 6B), in particular at higher PD values. Compared to WI-38 cells, BJ cells showed a much broader transition from partial cell cycle arrest to senescence, indicated by the increase of ageing markers and growth curves. We noticed for BJ cells other than in WI-38 cells, the number of p21 up-regulated cells is not a quantitative indicator of cell cycle arrested C cells: between PDs 30 and 55, this number is considerably higher than the calculated number of C cells (Figure 6B). Indeed, cell cycle arrest is regulated by CDKs (inhibited by p21); thus, p21 is only an indirect marker of cell cycle arrest. p21 seems to titrate CDKs differently in different fibroblasts.

**Figure 7:**
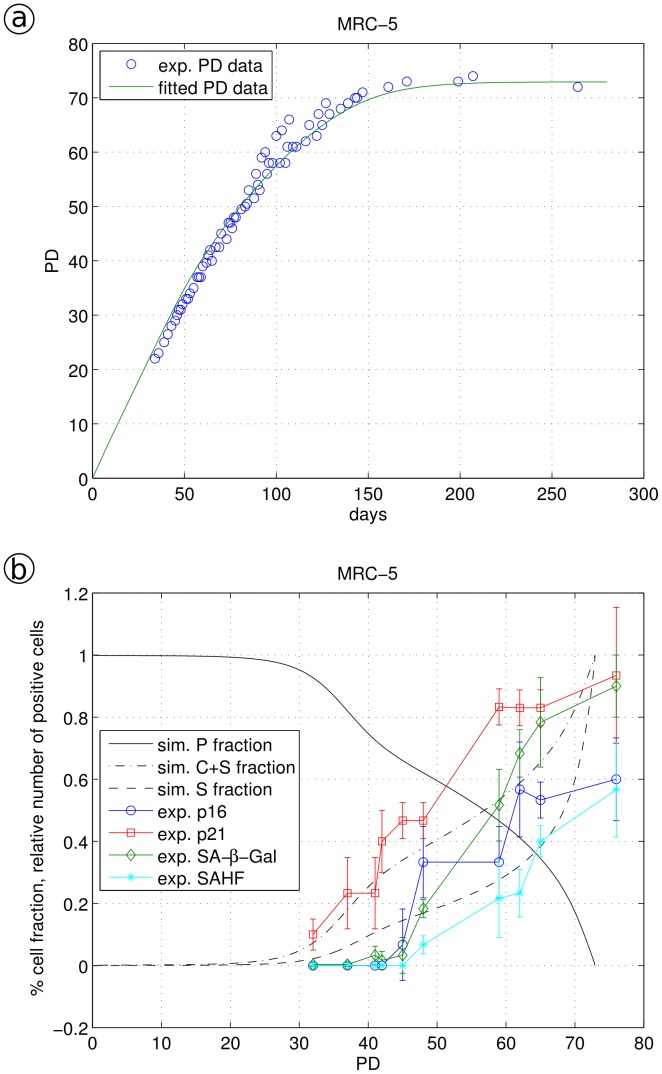
Simulation of MRC-5 fibroblast data. a, experimental data (circled) were fitted by model Eq. 3a–d using Eq. 4 as an expression for monotonically increasing stress γ; b, the fraction of proliferating cells P, cells showing a cell cycle arrest or a senescent phenotype (C+S) and solely the fraction of senescent cells S are shown together with the appearance of biomarkers. Biomarker values (p16, p21, SA-β-Gal and SAHF) were measured by immune-fluorescence as number of positive cells [49].

This is consistent with our observations in MRC-5 cells (Figure 7A, B). The up-regulation of senescence markers started already at low PDs: the number of p16 and SA-β-Gal positive cells started to increase at PD 48 (Figure 7B), immediately followed by SAHF formation. Applying γ (Eq. 4) with α = 0.004 and β = 0.0042 and the fitted values r = 0.489, f_1_ = 6.64, f_2_ = 5.99 and f_3_ = 0.26, we obtained a quantitative fit of the MRC-5 growth curve (Figure 7A) with a good fit for p21 at low PDs and an unsatisfying fit at higher PD values. Other than for WI-38 and BJ cells, the number of SA-β-Gal positive MRC-5 cells increased considerably while the cells still continued to grow (with only a slightly reduced rate). We were not able to find a set of model parameters which quantitatively describes both, the marker up-regulation as S cells as well as the growth curve. This suggests that in MRC-5 cells SA-β-Gal might increase not solely due to the cell transition into cellular senescence but additionally also due to other reasons, a finding consistent with a previous report [66]. Thus, identified by our model, SA-ß-Gal seems to be a good quantitative senescence marker in WI-38 and BJ cells but not in MRC-5 cells.

Although applying similar irradiation stress to WI-38 and MRC-5 cells, the cellular response is described by a roughly 3-fold lower γ value for WI-38 compared to MRC-5 cells. Obviously, WI-38 cells are more sensitive to irradiation than MRC-5 cells. This corresponds to WI-38 cells showing a rather sharp transition to senescence at low PD values. In contrast, BJ and MRC-5 fibroblasts undergo a considerably smoother transition towards cellular senescence at higher PD values. When in these three fibroblast cell lines, reactive oxygen species (ROS) production and telomere shortening accumulate to a similar extent, then the early and fast transition into senescence would indicate WI-38 cells indeed being more sensitive compared to the other two cell lines, consistent with published reports [60]. This agrees with results from the analysis of the f_1_, f_2_ and f_3_ parameter values and the γ expression influenced by α and β: for BJ cells, f_1_ and f_2_ are notably larger than for WI-38 cells, resembling a relatively high flux towards C cells and back to proliferating P cells. We interpret this as a higher maintenance workload for BJ than for WI-38 cells. In contrast, f_3_ is more than 2-fold smaller for BJ cells than for WI-38 cells resulting in a considerable delay for the BJ population to build-up a significant senescence cell fraction.

#### Influence from tissue origin and age

The observed cellular proliferation and the transition into senescence can vary depending on the tissue of origin and the age of the source tissue. Indeed, our data clearly indicate that for example cells from the same tissue source and the same donor age, but different gender (MRC-5 and WI-38 cell-lines) show clear differences in growth curves and transition to senescence. In order to determine if these observed differences are significant, we measured the experimental variance of our data by comparing the growth curves and the transition into senescence of primary human fibroblasts from the same tissue source (lung, MRC-5, WI-38), same donor age (MRC-5, WI-38 and BJ) and same or different gender (male: MRC-5, BJ; female: WI-38) as biological and technical replicates. When two aliquots of the same cell line but from different (commercial) sources were analyzed in parallel (biological replicates), we noticed small but significant differences between the growth curves of biological replicates for BJ, HFF, MRC-5 and WI-38 cells, but not IMR-90 cells. These small growth curve differences between two biological replicates, when fitted by our model, yielded in variations of transition rates between proliferating and cell cycle arrested cells (model parameters f_1_ and f_2_). In contrast, when cell-lines were separated into technical triplicates at early PD and analyzed in parallel, we observed no differences in their growth behavior: technical replicates showed excellent quantitative agreement between their growth curves yielding in identical model parameter values. Thus, the differences between the five human fibroblast cell-lines described above are significant.

#### Reduced growth rates can be explained by the presence of C and S cells with high r values

Experimentally determined cellular markers and our model identify the presence of cell cycle arrested and senescent cells in cell populations, as reported by others [60]. Thus, population growth might be determined by a constant (maximal) growth rate r for the proliferating P cells in the population, while the cell cycle arrested C and S cells do not contribute, i.e. the overall growth rate of the whole population is reduced relative to the maximal r value since it is a mixture of proliferating and non-proliferating cells. Fastest growth (r = 0.7, see Table 1) was observed for cancerous HeLa cells assumed not to be involved in cellular maintenance. We thus asked if we could explain the various growth rates presented in Table 1 by our model (Eq. 2a-b), now keeping r constant (r  =  r_max_ addressing the high value of r_max_ = 0.7 observed for HeLa cells) and fitting the transfer rate f_1_. Indeed, we obtained constant growth fitting the experimental values by a constant value for r and different values for f_1_, as given in Table 2. Thus, the observed variation in population growth could be explained by a constant high growth rate for the proliferating cells and for example by a varying amount of cell cycle arrested cells, which are assumed to be involved in cellular maintenance functions. This agrees with published observations on oxidative damage, for example for human fibroblast IMR-90 cells which divide faster under low level (3%) oxygen, with a doubling time 4–20% shorter, than for cells cultured under high (20%) oxygen, inducing more damage and thus requiring more maintenance [67]. However, different growth rates could also be explained by alternative cellular processes. The analysis of the biochemical origin of growth rate variation requires additional studies.

**Table 2:** Values of the parameter f_1_ in the model extension.

Species	Parameter *f_1_*
House mouse	2.00
Embryonic squirrel	3.02
Squirrel	8.31
Naked mole rat	3.83
Gerbil	2.28
Chinchilla	5.01
Rat	1.61
Chipmunk	4.84
Guinea pig	2.11
Muskrat	4.12
Hamster	1.88

f_1_ values for model extension with cell cycle arrest species. Same experimental data as in Table 1. For all simulations parameter r  =  r_max_ is set to 0.70 determined for HeLa cell growth rate and parameter f_2_ to 1.

#### Proliferation at constant stress depends on r relative to f_1_ f_3_/f_1_+ f_2_

Finally, we analytically investigated our model assuming a constant stress level (see Supplement S3), which is given, for instance, for constantly high (20%) oxygen levels. Our analysis shows that for r ≤ f_1_ f_3_/(f_2_+f_3_) the population is not able to grow, as the growth factor r is too small to overcome the cell cycle arresting and senescence effects. f_1_ and f_2_ have opposing effects: low f_1_ and high f_2_ promote cell growth, while high f_1_ and low f_2_ values result in senescence. While low f_3_ values result in a growing population, even high values of f_3_ can be overcome by sufficiently high proliferation rates r when the stress response function F is sufficiently small. Thus, regardless of the transition rate from C to S cells, the population can still grow under high C to S transitions as long as the C fraction of the population is low (mediated by low f_1_ and high f_2_ values). High f_3_ values could be important for populations living in environments that show high risks for rapid stress promoting events, where a fast transition into a senescent phenotype might be necessary for survival. In contrast, cells that rarely encounter dangerous events can afford rather low f_3_ values, as cell cycle arrested cells might well be rescued.

## Discussion

DNA damage is detected by cellular checkpoints. Checkpoints use a signaling mechanism either to stall the cell cycle until the damage has been repaired successfully or, if repair is unsuccessful, to target the cell for destruction via apoptosis. Under strong stress, some cell types cannot return to proliferation and enter the senescence state [47], [68], [69]. Here we present a quantitative model to simulate transient cell cycle arrest and cellular senescence. Next to the proliferating state P, we introduce a cell cycle arrested state C which is populated by rate f_1_ and returns to proliferation by rate f_2_. Once arrested, cells can become senescent (state S) with rate f_3_. In our model, the transition to the senescent state is considered as being irreversible (no back reaction). To some extent, the arrested state C is experimentally identified by the cell cycle inhibition marker p21, and the senescent state S by SA-β-Gal (and partly by p16). Our model combines these values of the different cellular states with the growth curves of these cells, quantitatively describing these.

Since the cellular response to stress is delayed, we introduced a stress response function F describing this behavior, as analogously used by [59]. F was designed to allow for a switch-like behavior distinguishing reversible cell cycle arrest as a consequence of low stress from irreversible arrest due to high or long-term stress. In its switched state, F cannot change back immediately after the stress source has decayed. Its nonlinear form is necessary to model two possible and well distinguishable steady states, describing here proliferating and strongly stressed cells. Moreover, we required F to enable a switch-like behavior from one state into the other in a rapid sigmoidal manner, once a critical point is exceeded. Switch-like behavior can be modeled also by other formulations (like, for example, by double negative feedback loops), however, choosing F in the form presented here offers the advantage of convenient analytical properties combined with a minimal amount of free parameters.

The stress itself can either be permanent (e.g. constantly high levels of oxygen), transient (due to, for example, induction of DNA damage by γ-irradiation pulses) or might increase with time (like telomere shortening). We therefore introduced a stress induction factor of the form γ  =  α+βt with a time-independent influence α and a time-dependent contribution βt.

A rigorous analytical analysis of the model for constant stress resulted in a relation between growth rate r and the transition rate quotient f_1_ f_3_/(f_2_+f_3_) which determines the fate of the cell population: with r smaller than the quotient, proliferation will stop while the population will continue to grow for r > f_1_ f_3_/(f_2_+f_3_).

We compared cellular data obtained for different stress levels in three different human fibroblast cell types. First, we induced two different levels of DNA damage by short and long term γ-irradiation, and successfully fitted our model by a single parameter set to the three growth curves. Then, we successfully fitted our model to cells growing into replicative senescence, regarded here to be mainly induced by oxidative stress and telomere shortening. Again, our model is able to explain the observed behavior by quantitatively describing the growth curves.

Applying our model to the experimental growth curves, we detected response differences between the WI-38 and the BJ or MRC-5 cell lines, consistent with published data on fibroblast line-specific properties [46], [60]. Such differences are not obvious from inspecting the experimental data, however, it could be well detected by our quantitative model analysis. WI-38 cells seem to be more sensitive to stress compared to MRC-5 cells since they reacted to smaller γ stress values. This is combined with a higher flux between P and C cells and a low transition into S cells for BJ cells, compared to lower transitions between P and C combined with a higher flux to S for WI-38 cells. This suggests considerable maintenance workload in BJ cells, which might explain its higher resistance to stress. Consistently, WI-38 cells show a faster transition to senescence at lower PD values than BJ and MRC-5 cells.

The observed differences in C (seen in BJ and MRC-5) and S cell fractions (seen in MRC-5) compared to p21 and SA-β-Gal marker levels, respectively, can be explained by the qualitative nature of these markers. It cannot be ruled out that their up-regulation has additional side-effects, which ultimately influence the growth behavior of the respective cell line. Therefore, our work highlights the need for further exploration of more specific cell cycle arrest- and senescence-specific biomarkers [9], [10].

Earlier, the influence of telomere shortening on cell proliferation was analyzed in network models of cell senescence [70]–[74]. Sozou and Kirkwood [72] applied a stochastic model for human diploid fibroblasts in which telomere reduction, oxidative stress linked to mitochondrial damage and nuclear somatic mutations were considered. Their model resulted in simulations that were in good agreement with data on intra-clonal variability in cell doubling potential published by [75]. Modeling the influence of telomere length on cellular senescence, Golubev et al. [73] conclude that telomere length decrease is a correlate of cell proliferation that, however, cannot alone account for senescence, instead also free radical damage influences have to be taken into account, consistent with statements by [72]. Portugal et al. [74] presented a stochastic growth model based on cell divisions in each time interval being a random process the probability of which decreases linearly with telomere shortening. The authors observed a good approximation of the qualitative growth of cultured human mesenchymal stem cells. In these models, theoretical parameters were not fitted to experimental data, in particular not to cellular growth curves. Lawless et al. [48] presented an analytical model fitting two cellular states, proliferation and senescence, to human fibroblast growth curves. Their model was successfully used to evaluate markers of cellular senescence. However, the model does not consider the intermediate state of transiently cell cycle arrested cells. When faced with mild stress, we observed here that a portion of the cells entered a temporary and reversible cell cycle arrest and not a senescence state, as indicated by the lack of SA-ß-Gal up-regulation (see Figure 3D). In a series of sophisticated analyses, B. Novak, J. J. Tyson and collaborators modeled the protein interaction network for the regulation of DNA synthesis and mitosis [76], [77]. Their approach provides a theoretical framework for the understanding of cell cycle regulation and presents increasingly complex models of the networks controlling cell growth and division. However, these authors did not model the cellular transition into senescence. Cellular senescence is maintained and reinforced by a DNA damage-ROS production feedback loop [78]. Passos et al. [78] presented a biochemically detailed stochastic model for this feedback loop on a single cell basis. Applying this model to our data, we were able to qualitatively simulate the cellular response to low as well as high irradiation. However, this model does not quantitatively compare cellular response to growth curve, a strength of our model.

In addition to entering the cell cycle arrested state C or the senescent state S as described here by our model, fibroblast cells may become apoptotic, enter the quiescent state or terminally differentiate. To address this question, specific markers for these cellular states should be quantified in order to further extend our current model.

## Supporting Information

Supplement S1 Bifurcation analysis of the stress response F(t).
(PDF)

Supplement S2 Sensitivity analysis.
(PDF)

Supplement S3 Analytical Analysis of the complete cellular senescence model.
(PDF)
