# Supplementary material for: Quantitative Model of Cell Cycle Arrest and Cellular Senescence in Primary Human Fibroblasts
Source: PLoS One. 2012 Aug 7;7(8):e42150. doi: 10.1371/journal.pone.0042150 (PMC3413708; doi:10.1371/journal.pone.0042150)
Supplement: Supplement S1 — Bifurcation analysis of the stress response F(t). (PDF) [file pone.0042150.s001.pdf]

Supplement S1 Bifurcation analysis of the stress response F(t)

$\frac{dF}{dt} = 0$

$F_1 : F_1 = 0$

$$F_2 : T\left(1 - \frac{F(t)}{K}\right) = \frac{F(t)}{1+F(t)^2} \quad \text{(S1.1)}$$

When is $T(1 - \frac{F(t)}{K})$ at a tangent to $\frac{F(t)}{1+F(t)^2}$?

$\frac{d}{dF}(T\left(1 - \frac{F(t)}{K}\right)) = \frac{d}{dF}\frac{F(t)}{1+F(t)^2}$

$$-\frac{T}{K} = \frac{1-F(t)^2}{(1+F(t)^2)^2} \quad \text{(S1.2)}$$

Substituting (S1.2) into (S1.1) yields

$$T - \frac{F(t)^2-1}{(1+F(t)^2)^2}F(t) = \frac{F(t)}{1+F(t)^2}$$

$$T = \frac{F(t)^3-F(t)+F(t)+F(t)^3}{(1+F(t)^2)^2}$$

$$T = \frac{2F(t)^3}{(1+F(t)^2)^2} \quad \text{(S1.3)}$$

Thus, a saddle-note bifurcation occurs if *T* increases and becomes greater than (S1.3). The current stable fixpoint is annihilated by the instable one. Increasing *F* further leads to a rapid change to the only stable fixpoint left, resembling bistable behavior.
