# Supplementary material for: Quantitative Model of Cell Cycle Arrest and Cellular Senescence in Primary Human Fibroblasts
Source: PLoS One. 2012 Aug 7;7(8):e42150. doi: 10.1371/journal.pone.0042150 (PMC3413708; doi:10.1371/journal.pone.0042150)
Supplement: Supplement S2 — Sensitivity analysis. (PDF) [file pone.0042150.s002.pdf]

Supplement S2 Sensitivity analysis

The sensitivity of each parameter *k* was approximated by fixating all parameters except for *k*, which was uniformly varied in the vicinity of 10% around its optimal value. Repeated 100 times for each parameter, this resulted in a set of 100 simulation runs per parameter *k*. The variance of the final population doubling (PD) values of these runs was further analyzed and interpreted as sensitivity measure to variation of parameter *k*.

The sensitivity s_k for variation of parameter *k* was calculated via

$$s_k = \frac{1}{n-1}\left(\sum_{i=1}^{n}(x_i - \bar{x})^2\right)^{\frac{1}{2}},$$

with n being the number of simulation runs and x_i the final PD value of simulation i.

This procedure was conducted using the BJ and WI-38 replicative senescence data and has been repeated for every parameter given by model Equations 3a-d and 4. The sensitivities resulting from the variation of single parameter values are shown in Figure S2.1.

Figure S2.1

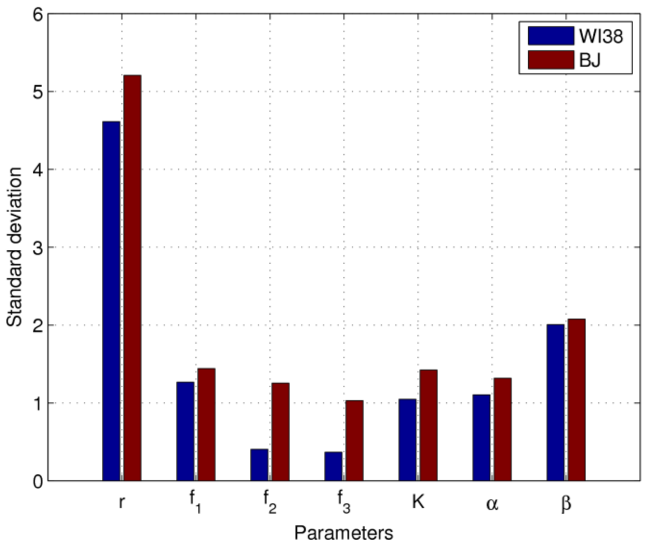

Sensitivity of model parameters as described by Eq. 3a-d and Eq. 4. The model is most sensitive to the variation of the growth rate r, resembling its particular importance for the maximal replicative capacity of cell cultures. Regarding the parameters f_{1-3}, BJ as well as WI-38 cells show qualitatively the highest sensitivity for P -> C transition, followed by its back transition (with rate f_2) and the C->S transition rate f_3. Interestingly, WI-38 cells are notably less sensitive to f_2 and f_3 variation than BJ cells. β shows a higher sensitivity than α, as it is a concentration dependent parameter, whereas concentration independent α is responsible for linearly accumulating long term stress.
