# Supplementary material for: Quantitative Model of Cell Cycle Arrest and Cellular Senescence in Primary Human Fibroblasts
Source: PLoS One. 2012 Aug 7;7(8):e42150. doi: 10.1371/journal.pone.0042150 (PMC3413708; doi:10.1371/journal.pone.0042150)
Supplement: Supplement S3 — Analytical Analysis of the complete cellular senescence model. (PDF) [file pone.0042150.s003.pdf]

Supplement S3 Analytical Analysis of the complete cellular senescence model

Assumption: Let $F(t)$ be constant: $f_1 F(t) \equiv \hat{f}_1$

The ODE system reduces to:

$$\dot{P} = rP - \hat{f}_1 P + f_2 C = (r - \hat{f}_1)P + f_2 C$$

$$\dot{C} = \hat{f}_1 P - f_2 C - f_3 C \qquad \text{(S3.1)}$$

$$\dot{S} = f_3 C$$

Observation: As the concentration of P increases with parameter r, no conservation relation can be retrieved from this system.

However, calculating the fractions $R_1 = \frac{P}{P+C+S}$, $R_2 = \frac{C}{P+C+S}$ and $R_3 = \frac{S}{P+C+S}$, yields the conservation relation:

$$R_1 + R_2 + R_3 = 1 \qquad \text{(S3.2)}$$

The population doublings are calculated as follows:

$$PD = log_2([P + C + S]) \qquad \text{(S3.3)}$$

By substituting (S3.1) into (S3.3) we derive:

$$\dot{P}D = log_2(P + C + S) \qquad \text{(S3.4)}$$

$$= \frac{1}{ln\,2} \frac{1}{P + C + S} (\dot{P} + \dot{C} + \dot{S}) \qquad \text{(S3.5)}$$

$$\overset{(1)}{=} \frac{r}{ln\,2} \frac{P}{P + C + S} = \frac{r}{ln\,2} R_1 \qquad \text{(S3.6)}$$

Thus, the time dependent concentration change of $\dot{P}D$ is zero, if r or R_1 is zero and greater than zero otherwise, since r, R_1 > 0. Consequently, R_1 is zero, if P is zero, implying that no proliferating cells are left to the population.

Moreover, applying the conservation relation (S3.2), it is sufficient to derive a solution for R_1 and R_2 in order to know the concentration fraction of R_3.

Isoclines

$$\dot{R}_1 = \frac{\dot{P}(P + C + S) - P(\dot{P} + \dot{C} + \dot{S})}{(P + C + S)^2}$$

$$= \frac{(r - \hat{f}_1)P}{P + C + S} + \frac{f_2 C}{P + C + S} - \frac{rP^2}{(P + C + S)^2}$$

$$= -rR_1^2 + (r - \hat{f}_1)R_1 - f_2 R_2 \qquad \text{(S3.7)}$$

$$\dot{R}_2 = \frac{\dot{C}}{P + C + S} - \frac{rPC}{(P + C + S)^2}$$

$$= \hat{f}_1 R_1 - (f_2 + f_3)R_2 - rR_1 R_2 \qquad \text{(S3.8)}$$

By setting (S3.7) and (S3.8) equal to zero we derive the following isoclines:

$$\dot{R}_1 = 0:$$

$$R_2 = \frac{r}{f_2} R_1^2 + \frac{\hat{f}_1 - r}{f_2} R_1 = y_1 \qquad \text{(S3.9)}$$

$$\dot{R}_2 = 0:$$

$$R_2(rR_1 + f_2 + f_3) = \hat{f}_1 R_1$$

$$R_2 = \frac{\hat{f}_1 R_1}{rR_1 + f_2 + f_3} = y_2 \qquad \text{(S3.10)}$$

Further analysis of (S3.9) and (S3.10):

$$y_1' = \frac{2r}{f_2} R_1 + \frac{\hat{f}_1 - r}{f_2} \qquad \text{(S3.11)}$$

$$y_1'' = \frac{2r}{f_2} \qquad \text{(S3.12)}$$

$$y_1' = 0:$$

$$2rR_1 + \hat{f}_1 - r = 0$$

$$R_1^0 = \frac{r - \hat{f}_1}{2r} \qquad \text{(S3.13)}$$

$y_1 = 0,\ y_2 = 0:$

$$R_1\left(\frac{r}{f_2}R_1 + \frac{\hat{f}_1 - r}{f_2}\right) = 0$$

$$y_1^{0_1} = 0, \qquad \text{(S3.14)}$$

$$\frac{r - \hat{f}_1}{f_2}\frac{f_2}{r} = y_1^{0_2} = \frac{r - \hat{f}_1}{r} \qquad \text{(S3.15)}$$

$$y_2^0 = 0 \qquad \text{(S3.16)}$$

$$\lim_{t \to \infty} y_2 = \frac{\hat{f}_1}{r} \qquad \text{(S3.17)}$$

It is now convenient to look into a graphical representation of y_1 and y_2. According to (S3.9), (S3.10) and (S3.13- S3.17) the graphs look as follows:

Figure S3.1: $r > \hat{f}_1$

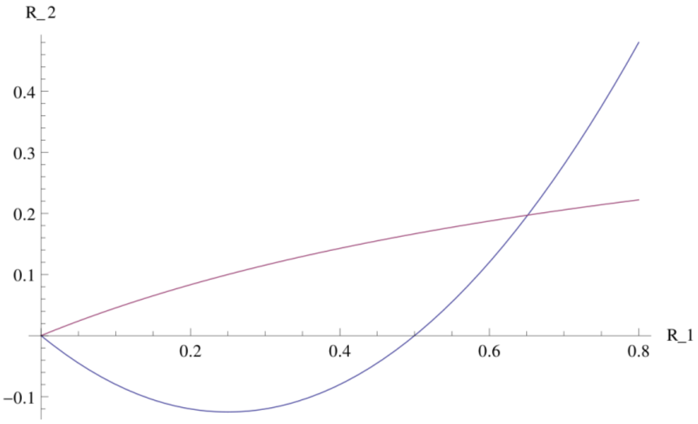

Figure S3.2: $r = \hat{f}_1$

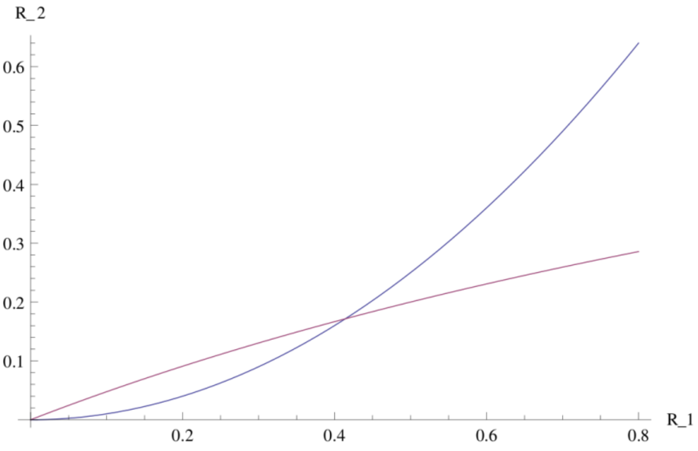

Figure S3.3: $r < \hat{f}_1$

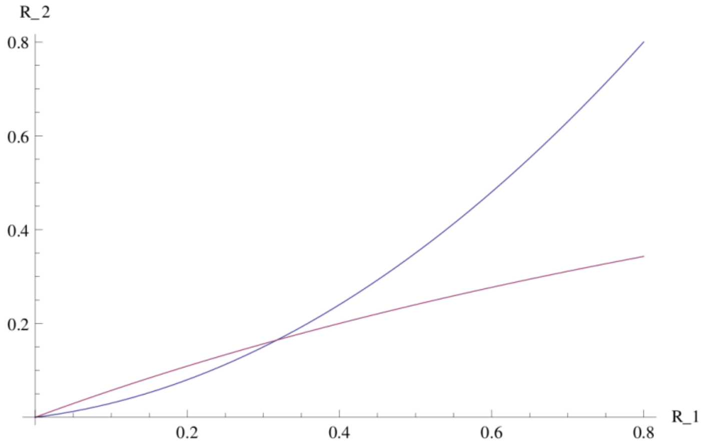

The parameter settings that relate to Figure S3.1 and S3.2 always yield a stable nonzero solution for R_1 and R_2 and thus $\dot{P}D \neq 0$. Now, case 3 (Figure S3.3) is of particular interest, as further decrease of r relative to $\hat{f}_1$ yields:

Figure S3.4: $r << \hat{f}_1$

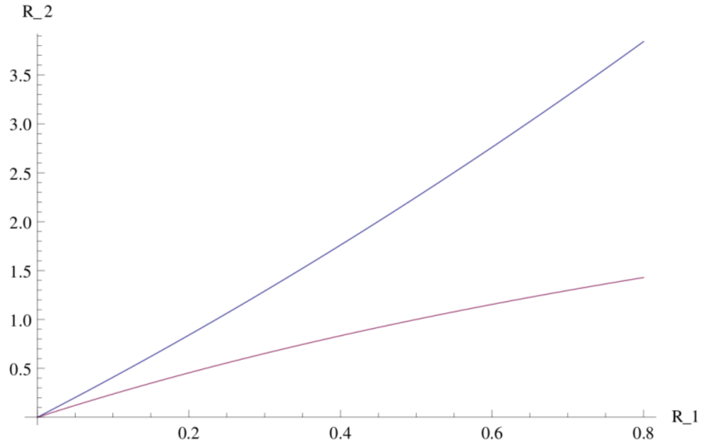

Parameter settings according to Figure S3.3, again yield a nonzero stable solution for R_1 and R_2, whereas in the case of Figure S3.4, the only stable solution for R_1 and R_2 is zero.

We derive this solution by computing:

$$y_1'(0) \geq y_2'(0) \tag{S3.18}$$

$$y_1'(0) = \frac{\hat{f}_1 - r}{f_2} \tag{S3.19}$$

$$\frac{\hat{f}_1(f_2 + f_3)}{(f_2 + f_3)^2} = y_2'(0) = \frac{\hat{f}_1}{f_2 + f_3} \quad \text{(S3.20)}$$

The fixed point at (0,0) in the (R_1, R_2) -plane is stable, if (S3.18) holds.

$$\frac{\hat{f}_1 - r}{f_2} \geq \frac{\hat{f}_1}{f_2 + f_3}$$

$$\hat{f}_1 f_3 \geq r(f_2 + f_3)$$

$$\hat{f}_1 \geq r\left(\frac{f_2}{f_3} + 1\right) \quad \text{(S3.21)}$$

$$r \leq \frac{\hat{f}_1 f_3}{f_2 + f_3} \quad \text{(S3.22)}$$

Thus, R_1 and consequently (S3.4) equals zero, if (S3.21) or (S3.22) holds, respectively, or is greater than zero otherwise.
